# exo-FISH: Protocol for detecting DNA breaks in repetitive regions of mammalian genomes

**DOI:** 10.1016/j.xpro.2023.102487

**Published:** 2023-08-06

**Authors:** Xanita Saayman, Emily Graham, Chin Wei Brian Leung, Fumiko Esashi

**Affiliations:** 1 Sir William Dunn School of Pathology, University of Oxford, Oxford, UK

**Keywords:** Cell Biology, Single Cell, Microscopy, Molecular Biology, *In Situ* Hybridization

## Abstract

Detecting DNA breaks in defined regions of the genome is critical to advancing our understanding of genome stability maintenance. Here, we present exo-FISH, a protocol to label exposed single-stranded DNA in defined repetitive regions of mammalian genomes by combining *in vitro* restriction enzyme digestion on fixed cells with fluorescence *in situ* hybridization (FISH). We describe steps for cell harvesting and fixation, slide treatments, and FISH probe hybridization. We then detail procedures for imaging and analysis.

For complete details on the use and execution of this protocol, please refer to Saayman et al. (2023).^1^

## Highlights

• Revealing DNA breaks in defined repetitive regions of human genomes in single cells
• Direct detection of DNA breaks, bypassing indirect markers or PCR amplification
• Multiplexing options for simultaneous assessment of different regions of the genome
• Adaptable to different cell lines, cell cycle stages, and regions of the genome

## Abstract (editor highlights)

Publisher’s note: Undertaking any experimental protocol requires adherence to local institutional guidelines for laboratory safety and ethics.

## Graphical abstract

**Figure:**
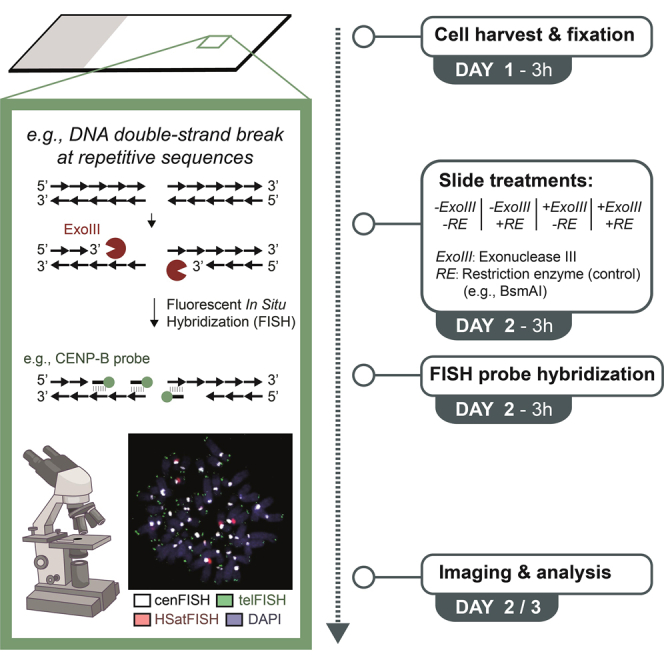

## Before you begin

1. exo-FISH is a protocol designed to assess the presence and relative abundance of spontaneous or condition-specific DNA breaks in defined repetitive regions of the genome. exo-FISH relies on Exonuclease III (ExoIII)-mediated digestion targeting either single-stranded DNA (ssDNA) or double-stranded DNA (dsDNA) breaks as initial substrates. ExoIII digests a single strand of dsDNA in the 3′ to 5′ direction to generate ssDNA tracts surrounding DNA breaks, which are then hybridized to fluorescent probes of complementary sequences. The degree of exposed ssDNA and, in turn, the degree of fluorescence intensity, is thereby proportional to the number of initial DNA breaks. By comparing the fluorescence responsiveness of different regions of the genome, users can infer the relative amount of DNA breaks present within defined repetitive regions.
2. In this work, we describe exo-FISH designed to simultaneously compare DNA breaks in three repetitive regions of the human genome, enabled by multiplexing fluorophores. First, the cenFISH probe is specific to higher order repeats (HORs) of human centromeres by targeting CENP-B binding sites (5′-ATTCGTTGGAAACGGGA-3′) and is a pre-designed commercially available probe (PNABio). Second, the telFISH probe is specific to telomere repeat sequences (5′-TAACCC-3′) of the human genome and is also a pre-designed commercially available probe (PNABio). Finally, the HSatFISH2/3 probe is a combination set specific to human satellite 2 and 3 (HSat2, HSat3) repeats, custom-designed using known HSat2 and HSat3 sequences (5′-TCGAGTCCATTCGATGAT-3′ and 5′-TCCACTCGGGTTGATT-3′, respectively). In all cases, probe sequences were aligned to the human reference genome (T2T-CHM13) to ensure specificity to the targeted region and verified experimentally by assessing probe localization on metaphase spreads (e.g., Figure 2). The probes are all composed of PNA, a synthetic analogue of DNA, for increased stability and resistance to nucleases and proteases, labeled with fluorophores. In the described protocol, we multiplexed Cy3, Cy5 and Alexa Fluor 488 fluorophores. Although alternative fluorophores are theoretically possible, we have not systematically compared fluorophores.
3. This protocol describes exo-FISH applied to mitotic hTERT-RPE1 cells, including validation using restriction enzyme digestions (BsmAI / Nt.BsmAI) as positive controls for first-time users. This protocol was used for exo-FISH optimization and validation, later applied to Saayman et al. 2023.^1^ This protocol can also be applied to non-mitotic (e.g., replicating, quiescent) cells, and has also been successfully applied to other human cell lines such as HCT116 and HeLa. exo-FISH should theoretically work in any organisms’ tissue culture cells upon appropriate optimization.

### Institutional permissions (if applicable)

No experiments were performed on live vertebrates or higher invertebrates - institutional permissions are not required.

### Reagent preparation

**Timing: 2–3 h**

4. Prepare 0.56% KCl Solution.
5. Prepare RNaseA Solution.
6. Prepare 70%, 95%, 100% EtOH.
7. Prepare 10% Blocking Reagent.
8. Prepare Hybridization Solution.
9. Prepare Hybridization Wash Buffer #1.
10. Prepare Hybridization Wash Buffer #2.
11. Prepare FISH Probe Stocks.
12. Prepare FISH Probe Solution.

## Key resources table

**Table:** 

REAGENT or RESOURCE	SOURCE	IDENTIFIER
**Chemicals, peptides, and recombinant proteins**		

Bovine serum albumin (BSA)	Sigma	Cat#A7906
S-trityl-L-cysteine (STLC)	Sigma	Cat#164739
Potassium chloride (KCl)	VWR	Cat# 26764.260
RNaseA	Sigma	Cat#R6513
Dulbecco’s Phosphate-Buffered Saline (PBS)	Sigma	Cat#D8537
Ethanol (EtOH)	Sigma	Cat#32221
Methanol (MeOH)	Sigma	Cat#32213
Glacial acetic acid (AA)	Thermo Fisher	Cat#10171460
BsmAI	NEB	Cat# R0529S
Nt.BsmAI	NEB	Cat#R0121S
Exonuclease III (ExoIII)	Promega	Cat#M1811
Maleic acid	Sigma	Cat#M0375
Blocking reagent	Roche	Cat#11096176001
Formamide	Merck	Cat#F9037
DAPI	BD Biosciences	Cat#D564907
ProLong Gold Antifade Mountant	Thermo Fisher	Cat#P10144

**Experimental models: Cell lines**		

hTERT-RPE1	ATCC	RRID:CVCL4388
HCT116	ATCC	RRID:CVCL0291
HeLa	ATCC	RRID:CVCL0030

**Software and algorithms**		

Olympus FV1000 software FV10-ASW v4.2	Olympus	RRID:SCR_014215 https://www.olympus-lifescience.com/en/downloads/detail-iframe/?0[downloads][id]=847249651
Fiji is Just ImageJ (Fiji) v2.0.0-rc-69/1.52p	N/A	RRID:SCR_002285 https://imagej.net/software/fiji/downloads
GraphPad Prism v8.4.3	N/A	RRID:SCR_002798 https://www.graphpad.com/features
Python v3.8.10	N/A	RRID: SCR_008394 https://www.python.org/downloads/

**Other**		

FISH PNA probe against centromere higher order repeats (HORs) (Cy3) 5′ ATTCGTTGGAAACGGGA 3′	PNABio	Cat#F3009
FISH PNA probe against telomeres (Cy5) 5′ CCCTAACCCTAACCCTAA 3′	PNABio	Cat#F1003
FISH PNA probe against human satellite 2 repeats (HSat2) (Alexa fluor 488) 5′ TCGAGTCCATTCGATGAT 3′	Custom order (PNABio)	N/A
FISH PNA probe against human satellite 3 repeats (HSat3) (Alexa fluor 488) 5′ TCCACTCGGGTTGATT 3′	Custom order (PNABio)	N/A
Glass slides (75 mm × 25 mm)	VWR	Cat#631-1553
Glass coverslips (22 mm × 64 mm)	VWR	Cat#631-0880
Coplin jars	Sigma	Cat#S5516
Incubation chamber (dark, humid)	Custom	N/A
FV1000 Fluoview laser scanning microscope	Olympus	N/A

## Materials and equipment

**Table:** Hybridization Solution

Reagent	Final concentration	Amount
Tris-HCl pH 7.2 (1 M)	10 mM	10 μL
Formamide (100%)	70%	700 μL
Blocking Reagent (10%)	0.5%	50 μL
ddH_2_O	N/A	240 μL
**Total**	**N/A**	**1 mL**

**CRITICAL:** Formamide is a suspected carcinogen with demonstrated toxic reproductive effects. Limit exposure by only handling in a chemical fume hood with full protective gear. Formamide must be disposed of appropriately to prevent environmental contamination.

***Note:*** Here, and throughout the exo-FISH protocol, double-distilled water (ddH_2_O) was used.

**Table:** Hybridization Wash Buffer #1

Reagent	Final concentration	Amount
Tris-HCl pH 7.2 (1 M)	10 mM	0.5 mL
Formamide (100%)	70%	35 mL
BSA (10%)	0.1%	0.5 mL
ddH_2_O	N/A	14 mL
**Total**	**N/A**	**50 mL**

***Note:*** 10% BSA stock (made fresh each time) must be made in ddH_2_O in advance and fully dissolved before adding to formamide. Hybridization Wash Buffer #1 can be re-used 2–3 times if users are using the same probe combinations, stored in 4°C between uses and for up to a month.

**Table:** Hybridization Wash Buffer #2

Reagent	Final concentration	Amount
Tris-HCl pH 7.2 (1 M)	100 mM	15 mL
NaCl (5 M)	150 mM	4.5 mL
Tween-20 (100%)	0.08%	120 μL
ddH_2_O	N/A	130.38 mL
**Total**	**N/A**	**150 mL**

***Note:*** Hybridization Wash Buffer #2 can be made in advance and stored at room temperature (20°C–25°C) for up to a month.

### Other solutions

• **5 μM S-trityl-L-cysteine (STLC):** 5 μM STLC dissolved in 100% DMSO.

***Note:*** 5 μM STLC can be made in advance, and aliquots stored at −20°C.

• **0.56% KCl Solution:** 0.56% (w/v) potassium chloride (KCl) in ddH_2_O.

***Note:*** 0.56% KCl can be made in advance and stored at room temperature (20°C–25°C).

• **Fixative Solution (3:1 MeOH:AA):** 3:1 volume methanol to glacial acetic acid (e.g., 15 mL 100% MeOH with 5 mL glacial acetic acid).

***Note:*** Fixative Solution must be made fresh directly before fixation and not stored.

**CRITICAL:** Both methanol and acetic acid are flammable as liquid and vapors, and harmful if swallowed, inhaled or in contact with skin. Acetic acid can cause severe skin burns and eye damage. As such, Fixative Solution should be made and handled in a chemical fume food with full laboratory protective gear.

• **RNaseA Solution:** 0.5 mg/mL RNaseA diluted in PBS, made fresh each time.
• **75% / 95% EtOH:** 75% / 95% (v/v) EtOH in ddH_2_O.

***Note:*** Can be made in advance and stored at room temperature (20°C–25°C) for up to a month.

• **Maleic Acid Buffer:** 100 mM maleic acid in 150 mM NaCl, brought to pH 7.5 with 1 M NaOH.
• **10% Blocking Reagent:** 10% (w/v) Blocking Reagent in Maleic Acid Buffer.

***Note:*** Fully dissolving Blocking Reagent may take some time and require heating in a heating block, water bath or microwave. 10% Blocking Reagent can be made and stored at 4°C and used for up to a month.

• **FISH Probe Stocks:** Dried FISH probes (from PNABio) should be made to 100 μM in formamide and fully dissolved by heating for 10 min at 60°C with occasional vortexing.

***Note:*** Stocks can be aliquoted (10 μL) and stored at −80°C for at least several months. Working aliquots can be kept at 4°C in the dark for up to 2 weeks.

• **FISH Probe Solution:** 1:200 dilution of 100 μM stocks diluted in Hybridization Solution. Vortex dilutions well to distribute evenly.

## Step-by-step method details

### STEP 1: Cell harvest, fixation, and preparation of (metaphase) spreads – Day 1

**Timing: 3 h sample processing excluding pretreatment and slide drying, ∼24 h inclusive**

Mitotic cells are harvested following experimental treatment, fixed, and spread on glass slides.

**Figure 1:**
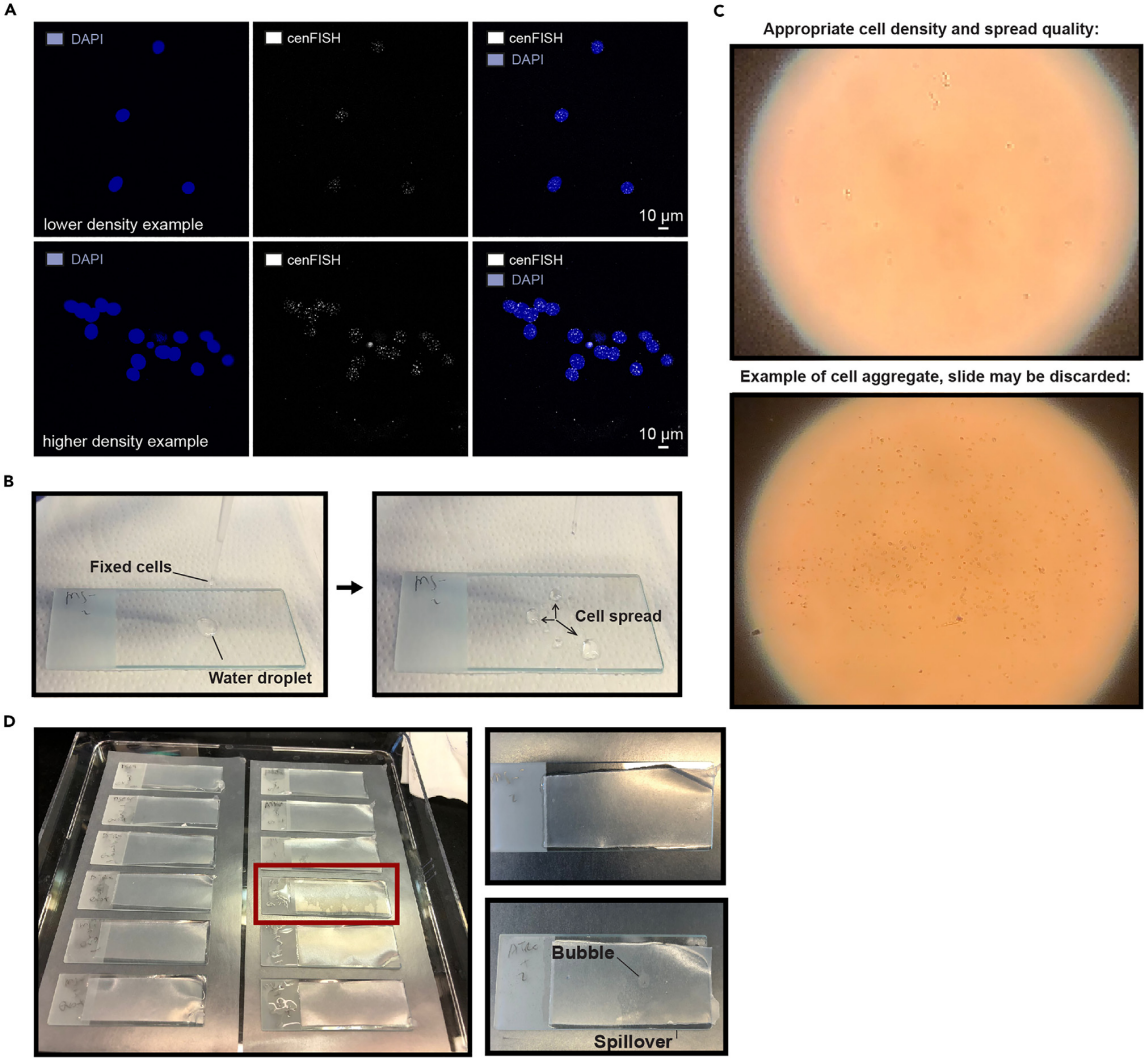
Protocol examples (A) Examples of how cell density can influence FISH staining intensity. Images were taken from the same slide using the same settings, at 60× magnification. Scale bar represents 10 μm. (B) Example of cell spreading when placing fixative-containing drop on water droplet on glass slides. (C) Example of cell spreads visualized with a light microscope at 10× magnification. Top: a slide with appropriate cell density and homogenous distribution, often with a lighter cell outline. Bottom: a slide with a large cell aggregate, as often marked by darker cell outlines. (D) Left: image of slides undergoing ExoIII treatment with parafilm strips to equally distribute treatment across slide. Example of a liquid ‘spillover’ highlighted in red. Right: comparison of appropriate liquid distribution (left) and bubbles / spillovers (right).

***Note:*** Cells should be actively replicating to enrich for mitotic cells, and so should be seeded far below confluency, to be ∼2–4 million cells per 10-cm dish, or ∼25%–50% confluency, at the point of collection. Multiple 10-cm dishes per experimental condition can be used if mitotic cell yields are low.

***Note:*** Equal cell numbers between experimental conditions as well as homogenous cell distributions are very important, as cell density impacts fluorescence intensity and cell aggregates display atypical fluorescence signals (see Figure 1A for an example). While this is not an issue for more classic localization applications of FISH, the quantitative nature of exo-FISH requires extra attention to this detail. As such, cells are counted to maintain constant cell numbers between experimental conditions, well homogenized during harvesting, and kept at low concentrations throughout fixation and washes. In addition, prepared slides should be inspected using a light microscope after making spreads to disregard slides with non-homogenous cell distributions or numbers.

***Note:*** Please see the troubleshooting section, problem 1 for troubleshooting options related to this step.

1. Treat cells cultured in 10-cm dishes with 5 μM S-trityl-L-cysteine (STLC) in complete media for >5 h to enrich for mitotic cells.

***Alternatives:*** Any reagent enriching for mitotic cells (e.g., colcemid, Roche cat. #10295892001) can work in place of STLC, but we have primarily performed exo-FISH using STLC for mitotic enrichment.

2. Aspirate 90% of the remaining media and perform mitotic shake-off to dislodge mitotic cells. Collect supernatant in a 15-mL conical tube.

***Note:*** Mitotic shake-off – mitotic cells are isolated by banging plates ∼10 times with force, against your palm or a hard surface. Inspection under a light microscope should reveal that most of the rounded (mitotic) cells are dislodged.

***Note:*** If not using mitotic cells, cells can be harvested by trypsinization (e.g., with TrypLE) cells for 3–5 min at 37°C, followed by collection in complete media or PBS with 1% BSA.

3. Wash plate(s) with PBS to collect any remaining mitotic cells and add to 15-mL conical tube.

***Alternatives:*** Any 15-mL conical tube can theoretically be used, but we used ThermoFisher Cat. No. 12604013 for this protocol.

4. Centrifuge 4 min, 500 x*g* at room temperature (20°C–25°C).

***Note:*** Room temperature (20°C–25°C) centrifugation is used throughout the protocol to prevent excessive cell aggregation.

5. Aspirate media and resuspend pellet in 1 mL PBS.
6. Count cells for each experimental condition.
7. Centrifuge 4 min, 500 x*g* at room temperature (20°C–25°C).
8. Wash pellets in PBS, diluting to ∼1 million cells per 1 mL of PBS.
9. Move 200 μL cell suspension to a fresh 15-mL tube (∼200 K cells).
10. Gradually add ∼10 mL of 0.56% KCl per 200 K cells to the side of the tube.
11. Invert tube gently 4 times to mix well.
12. Incubate for 20 min at room temperature (20°C–25°C) on a rocking platform shaker at low speed.

***Note:*** We used the Cole-Parmer Gyro Rocking Shaker (mini, 120 V) at 20 rpm. However, any appropriate rocking shaker can be used.

13. Centrifuge 5 min, 300 × *g.*
  a. During this time, prepare fresh 3:1 MeOH:AA Fixative Solution.
14. Aspirate the supernatant, leaving ∼100 μL liquid. Pipette up and down very gently to resuspend cell pellet.
15. Gradually add 1 mL fixative (3:1 MeOH:AA) dropwise on the side of the tube using a P1000 micropipette, while gently shaking the tube. Gently tap the tube to mix.
16. Add an additional 9 mL 3:1 MeOH:AA (10 mL total per 200 K cells), while gently shaking the tube.
17. Incubate for 20 min at room temperature (20°C–25°C) on a rocking shaker at low speed.
18. Centrifuge 5 min, 300 × *g.*
19. Aspirate the supernatant, leaving ∼100 μL liquid per 200 K cells. Pipette up and down very gently to resuspend cell pellet.

***Note:*** When aspirating, we use a standard to indicate the volume corresponding to ∼100 μL. It is important that remaining volumes are constant between experimental conditions.

20. Drop 10 μL of cells (such that ∼20 K cells are dropped per slide) onto glass slides on to the middle of a 15 μL ddH_2_O drop.

***Note:*** Repulsion between the fixative and ddH_2_O will cause cells to disperse more evenly. It helps to expel the 10 μL from the pipette tip until it forms a hanging drop, and then gently placing the hanging drop directly onto the center of the water droplet. See Figure 1B for an example.

***Note:*** Two well-separated spreads can be made per slide to test different experimental conditions on the same slide.

21. Inspect slides with a light microscope to ensure consistent cell densities and distributions between experimental conditions. Discard aberrant slides.

***Note:*** When choosing slides with appropriate cell densities and distributions, we look for slides with homogenously spread cells, often with lighter cell outlines. Slides with excessive cell aggregates are identified by having high cell densities or clusters of cells surrounding empty regions, often with darker cell outlines. See Figure 1C for examples.

***Note:*** We typically make at least twice the number of required slides, to ensure having enough slides with consistent cell densities and spreads.

22. Dry slides overnight (12–24 h) at room temperature (20°C–25°C), protected from light.

**CRITICAL:** All steps from this point should be performed while protected from light (e.g., covered coplin jars, covered incubation chambers, etc.). This is to avoid UV exposure and minimize induction of DNA breaks.

**Pause point:** After creating cell spreads, it is possible to store slides at −20°C for several days, up to a month. However, ExoIII digestion becomes less efficient with longer storage periods, and so it is not recommended to store slides for more than a day or two.

### STEP 2: Slide treatments and FISH probe hybridization – Day 2

**Timing: 6–7 h**

Slides are treated with RNaseA to remove RNA and prevent probe binding to RNA instead of DNA. Slides are then further treated to perform (optional) restriction enzyme digestion and expose ssDNA surrounding DNA breaks through ExoIII digestion. FISH probe hybridization then fluorescently labels exposed ssDNA.

***Note:*** This protocol includes BsmAI / Nt.BsmAI positive control restriction enzyme digestions (steps 29–32). These may be skipped once the user is familiar with the protocol.

***Note:*** Please see the troubleshooting section, problems 2, 3, and 4 for troubleshooting options related to this step.

23. Rehydrate slides in PBS for 5 min at room temperature (20°C–25°C) in coplin jars.
24. Dab slide edges on tissues to remove excess PBS and place slides with cells facing upwards on parafilm.
25. Gently add 500 μL 0.5 mg/mL RNaseA in PBS dropwise across the slide and cover with a parafilm strip.

***Note:*** Parafilm strips are cut to slightly smaller dimensions than the size of the slide, ∼1–2 mm smaller on each side, and laid on top, taking care to prevent any bubble formation.

26. Incubate at 37°C for 10 min in a humidified incubation chamber.
27. Wash slides in PBS for 5 min at room temperature (20°C–25°C) in coplin jars placed in the middle of the platform on a rocking platform shaker set to low speed.
28. Dab slide edges on tissues to remove excess PBS and dry the bottom of the slides to remove any moisture. Place slides with cells facing upwards on fresh parafilm.

**CRITICAL:** Any liquid on the bottom of the slides may cause the restriction enzyme-containing solution to spill under the slide, preventing equal treatment between slides. As such, slide bottoms should be well-dried before placing on parafilm. See Figure 1D for examples.

29. Add 200 μL 1× CutSmart containing either 0 or 1 total units BsmAI or Nt.BsmAI to each slide in order to induce double-stranded or single-stranded DNA breaks, respectively. Cover with a parafilm strip, ensuring no liquid spillover, equal distribution across the slide, and no bubble formation.

***Note:*** BsmAI and Nt.BsmAI were chosen as positive controls for cenFISH probes based on their recognition sequence abundance in human centromeres (∼900 bp between recognition sites, on average). As a positive control, the user should identify restriction enzymes appropriate to the FISH probe they are using. This can be done by assessing the distribution and frequency of restriction enzyme digestion sites in their region of interest using any standard cloning software (e.g., SnapGene).

30. Incubate at 37°C for 60 min in a humidified incubation chamber.
31. Wash slides in PBS for 5 min at room temperature (20°C–25°C) in coplin jars on a rocking platform shaker at low speed.
32. Dab slide edges on tissues to remove excess PBS and dry the bottom of the slides to remove any moisture. Place slides with cells facing upwards on fresh parafilm.

**CRITICAL:** Any liquid on the bottom of the slides may cause the ExoIII-containing solution to spill under the slide, preventing equal treatment between slides. As such, slide bottoms should be well-dried before placing on parafilm. See Figure 1D for examples.

33. Add 200 μL of 0 mU/μL or 200 mU/μL ExoIII solution to each slide, diluted in 1× buffer supplied by the manufacturer. Cover with a parafilm strip, ensuring no liquid spillover, equal distribution across the slide, and no bubble formation.

***Note:*** A condition without ExoIII, hereafter referred to as -ExoIII (i.e., 0 mU/μL), must be included in every experiment as a negative control, to account for differences in baseline FISH probe hybridization levels.

***Note:*** The ExoIII concentration should be optimized for each cell line to identify optimal signal ratios between +ExoIII and -ExoIII samples. If ExoIII concentrations are too high, it may be difficult to visualize -ExoIII samples without saturating fluorescent signals in the +ExoIII samples. On the other hand, if ExoIII concentrations are too low, it may be difficult to detect any differences between +ExoIII and -ExoIII samples. As such, we recommend trying 0, 50, 100, 200, and 500 mU/μL ExoIII in preliminary experiments.

34. Incubate at 37°C for 30 min in a humidified incubation chamber.
35. Wash slides in PBS for 5 min at room temperature (20°C–25°C) in coplin jars on a rocking platform shaker at low speed.
36. Wash slides in ethanol series: 5 min each in 70% EtOH, 95% EtOH, 100% EtOH in coplin jars on a rocking platform shaker at low speed.
37. Air dry slides at room temperature (20°C–25°C) for at least 1 h.

**Pause point:** Slides can be stored in the dark at this point until the following day or can be proceeded with after fully drying (∼1 h).

38. Add 200 μL FISH Probe Solution (1:200 of stock, diluted in Hybridization Solution) to dried slides and cover with a parafilm strip, ensuring no liquid spillover, equal distribution across the slide, and no bubble formation.

***Note:*** We have successfully used up to three FISH probes in the same solution (e.g., Cy3-CENPB, Cy5-Tel, A488-HSat2/3) without issues or fluorescence bleed-through.

39. Incubate for 1.5 h in the dark, at room temperature (20°C–25°C), in a humidified incubation chamber.
40. Transfer slides to coplin jars and wash in Hybridization Wash Buffer #1 for 15 min at room temperature (20°C–25°C) on a rocking platform shaker at low speed.
41. Transfer slides in Hybridization Wash Buffer #2 for 5 min at room temperature (20°C–25°C) on a rocking platform shaker at low speed.
42. Wash slides in Hybridization Wash Buffer #2 containing 0.5 μg/mL DAPI for 5 min at room temperature (20°C–25°C) on a rocking platform shaker at low speed.
43. Wash slides in Hybridization Wash Buffer #2 for 5 min at room temperature (20°C–25°C) on a rocking platform shaker at low speed.
44. Wash slides in ethanol series: 5 min each in 70% EtOH, 95% EtOH, 100% EtOH.
45. Air dry slides for ∼20 min, until no liquid remains on the slide surface.
46. Cover glass slides with 22 mm × 64 mm coverslips using ∼30 μL ProLong Gold Antifade Mounting media. Wait for mounting media to dry (at least 3 h) before proceeding to Imaging and Analysis (STEP 3).

**Pause point:** Slides can be stored short-term at 4°C or long-term at −20°C prior to imaging. We have not tested how long slides can be stored without losing fluorescence, but they can be stored for at least 1 month.

***Alternatives:*** Any appropriate mounting media can be used in place of Prolong Gold (e.g., Millipore MOWIOL® 4–88 Reagent, cat. #475904).

### STEP 3: Imaging and analysis

47. Image slides.

**CRITICAL:** We have observed that FISH signal intensities can have local differences across the slide, so it is important to take images throughout the slide. In addition, it is critical to avoid imaging aggregates given their abnormal FISH staining patterns. Cell selection should be done by DAPI staining. We typically image at least 30 cells per condition. At 60×–100×, this is typically 2–5 cells per image, or ∼10 images, taken from throughout the slide, per condition.

**CRITICAL:** Since fluorescence intensities will be quantified and compared to other experimental conditions, ensure that the laser settings are set such that there is no saturation in any of the channels or conditions.

48. Image analysis (see suggested approach in the ‘quantification and statistical analysis’ section below).

## Expected outcomes

**Figure 2:**
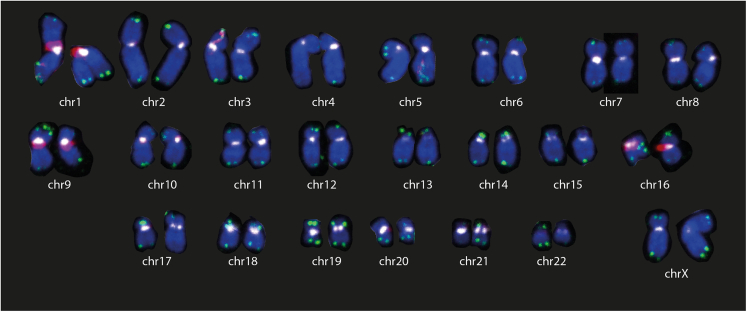
Expected FISH probe localization using cenFISH, telFISH and HSat FISH probes Karyogram depicting FISH probe hybridization patterns. FISH probes against higher-order-repeat (HOR)-associated centromere repeats (cenFISH, white), telomere repeats (telFISH, green), and human satellite 2 and 3 repeats (HSatFISH, red) are depicted. This figure is adapted from Saayman et al.^1^

If the protocol is successful, the user should expect to obtain FISH signal intensities localizing to appropriate regions of the genome (e.g., Figure 2), which is easily visualized in mitotic cell spreads.

**Figure 3:**
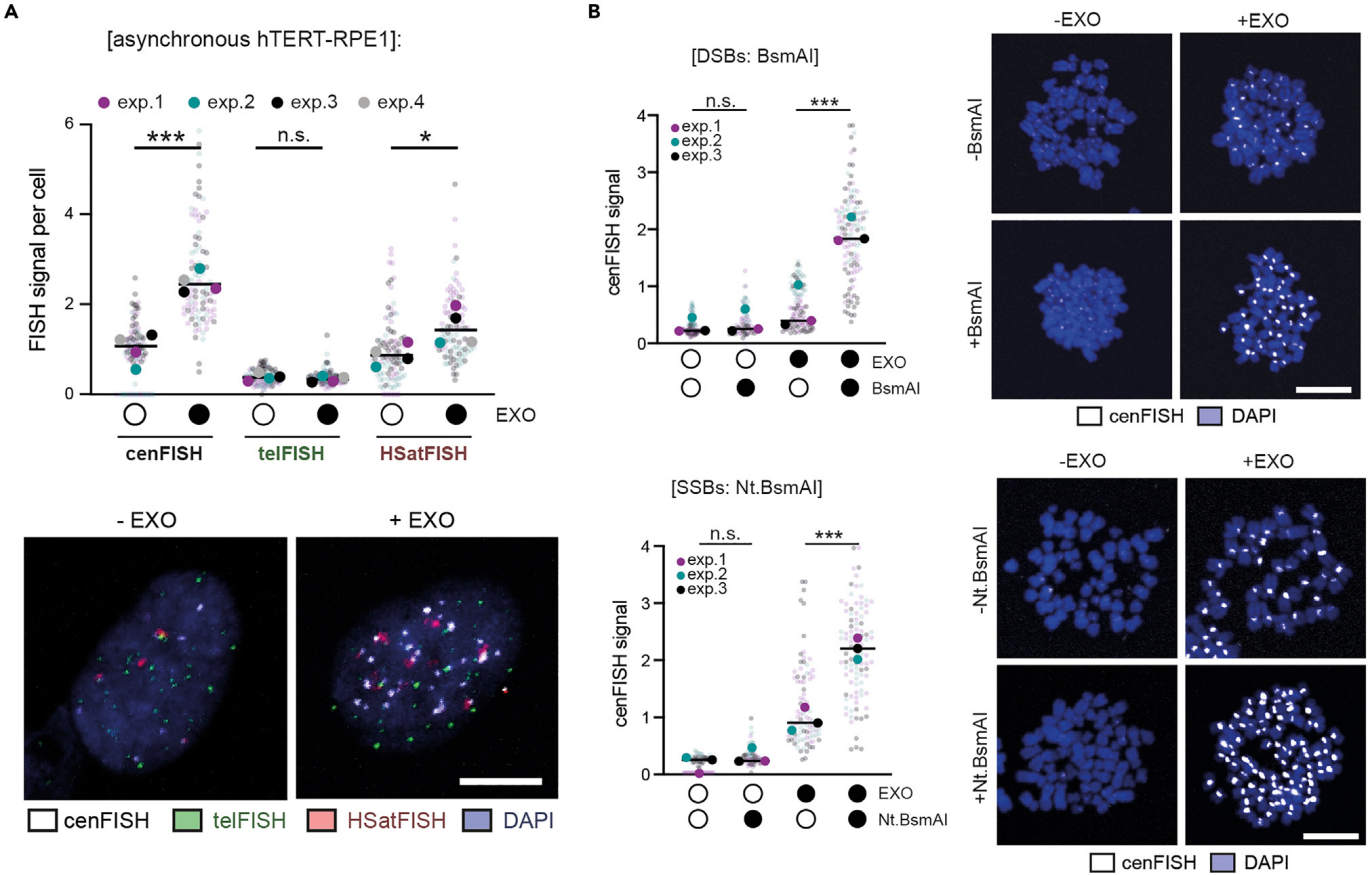
Expected outcomes of exo-FISH (A) Quantification and representative images of exo-FISH applied to asynchronous hTERT-RPE1 cells with and without ExoIII (EXO) treatment, using cenFISH, telFISH and HSatFISH probes. (B) Quantification and representative images of exo-FISH applied to mitotic hTERT-RPE1 cells arrested in mitosis using STLC. Double-stranded breaks (DSBs) or single-stranded breaks (SSBs) were induced by restriction enzyme digestion using BsmAI or Nt.BsmAI, respectively. At least 30 cells were imaged per experimental condition. The medians of each experimental condition were used to perform a two-sided unpaired t-test (∗p < 0.05, ∗∗p < 0.01, ∗∗∗p < 0.005). Filled and empty circles indicate presence and absence, respectively. Scale bar represents 10 μm. This figure is adapted from Saayman et al.^1^

After quantifying FISH signal intensities per cell, the expected results are increases in FISH fluorescence intensities upon ExoIII treatment, proportional to the number of DNA breaks in the given region. In our case, we observed strong increases in cenFISH signals, modest increases in HSatFISH signals, and little to no increases in telFISH signals upon ExoIII treatment in unperturbed conditions (as shown for asynchronous hTERT-RPE1 cells in Figure 3A and described in Saayman et al. 2023).

In addition, if using Nt.BsmAI / BsmAI positive controls, there should be a further FISH signal intensity increase upon restriction enzyme pre-treatment (which has only been tested for cenFISH, as in Figure 3B). Nt.BsmAI / BsmAI pre-treatment should not influence the -ExoIII control.

## Quantification and statistical analysis

Here, we provide a basic description of our chosen method for data quantification. This focus-based analysis was developed to account for background fluorescence signal and extract foci data, capturing both focus size and intensity attributes. However, any other appropriate method of data analysis can be applied according to the user’s interests.

### Fiji-based focus data extraction

If multiple slices were imaged, Z-project slices:

>run(”Z Project ...” , ”projection=[Sum Slices ]”);

Split channels:

>run(” Split Channels”);

Segment cells according to DAPI channel to separate cells as regions of interest (ROIs), after filling in DAPI holes to capture full cells:

***Note:*** for mitotic cells, manual cell definition is required since chromosomes are spatially separated.

>run(”8−bit ”);

>run(”Auto Threshold”, ”method=Li white”);

>run (” Convert to Mask”);

>run(” Dilate ”);

>run(”Fill Holes”); run (” Erode ”);

>run(”Analyze Particles ...” , “size=30-Infinity display exclude clear summarize add”);

For each ROI, detect cenFISH, telFISH, HSatFISH foci according to user-defined threshold (based on fluorescence intensities – this can be channel-specific but must be kept consistent throughout image analysis). Then, draw boxes (__x__ pixels, depending on resolution) around each focus and export data (e.g., for focus i, in channel 3 positioned at x, y with a 10 × 10 pixel box):

>run(”Find Maxima...” , ”prominence=400 output=List ”);

>makeRectangle(x-5, y-5, 10, 10);

>run(”Duplicate...”, ”title=i” duplicate channels = 3”);

>saveAs(”Text Image”, dir+”i.txt”);

### Foci analysis, visualization, and statistical analysis

First, using Python / R / other analysis software, import all foci data of a given cell. Background signals can then be estimated for each focus using the median perimeter signal readings and multiplying by the box size. Second, the net focus signal can be calculated by subtracting the estimated background signal from the sum of the focus signal intensity. Taking the mean/median of all net (i.e., background-subtracted) foci signal intensities for a given cell yields ‘FISH signal per cell’, as is shown in Figure 3. Finally, this is repeated for all cells of all conditions and can be plotted directly or exported for plotting with GraphPad Prism. Once at least three biological replicates have been acquired, the relevant statistical test can be performed on the means/medians of the biological replicates to determine statistical significance.

## Limitations

From our experience using exo-FISH, we advise that the following points should be kept in consideration when planning and executing exo-FISH experiments. First, exo-FISH can have variable results due to the fluorescence artefacts related to cell densities described above, and so biological replicates are absolutely essential before reaching conclusions. Second, exo-FISH uses ExoIII, which acts on both single- and double-stranded breaks as initial substrates. As such, it is not possible to discern between single- and double-strand breaks in genome regions of interest. Third, ExoIII has reported alternative activities including RNaseH, 3′-phosphatase and apurinic/apyrimidinic (AP)-endonuclease activity,^2^ and so additional experiments are required to support findings of DNA breaks in a region of interest. Finally, ExoIII may begin digesting from DNA breaks in the vicinity of FISH probe targets, and it is currently unknown to what extent ExoIII digests DNA away from initial break sites. This may create a false appearance of DNA breaks within the region of interest. Along the same lines, it is theoretically possible that high densities of DNA breaks can lead to ‘over-digestion’ due to reciprocal digestion of adjacent DNA breaks, such that ssDNA tracts are completely removed, and FISH probes have no complementary sequences to bind to. As such, extended (>30 min) or high concentrations (>500 mU/μL) ExoIII treatment are advised against.

## Troubleshooting

### Problem 1

Inconsistent cell densities and/or distributions (related to STEP 1).

### Potential solution

FISH fluorescence signal intensities can depend on cell density and the quality of cell spreads. Depending on the cell line and experimental condition, getting consistent, high-quality cell spreads can be challenging. If the user is unable to get consistent cell densities between experimental conditions, it is possible to normalize signal intensities to neutral / reference FISH probes (e.g., telFISH, in our case) to control for these effects at a given region of interest (e.g., cenFISH, in our case). If the user is struggling to get high-quality cell spreads, several existing resources are available for reference on improving the quality of cell spreads.^3^

### Problem 2

No or low FISH signals (related to STEP 2).

### Potential solution

If FISH signals are completely absent, we recommend first ensuring that FISH probes are functioning (i.e., able to hybridize to the appropriate region of interest) by performing standard FISH hybridization protocols under DNA denaturing conditions (heating slides and probes at 80°C for 10 min before the hybridization step). Alternatively, the user can assess whether the FISH probes are correctly labeled with the appropriate fluorophore by running them on a 2% agarose gel and visualizing gels with a fluorescence gel imager. The user can also verify that the given probe sequence is present in the respective cell line if a reference genome is available. If FISH signals are present but not clearly visible for a given region of interest, one possibility is that cell densities are too low. Increasing cell densities on slides (e.g., dropping 50 K cells per spread) for all conditions may improve the strength of FISH signal intensities.

### Problem 3

No FISH signal responsiveness to ExoIII (related to STEP 2).

### Potential solution

The responsiveness to ExoIII should be proportional to the amount of DNA breaks present in a given region, and so the absence of ExoIII responsiveness could be due to biological or technical reasons. If the user is not seeing ExoIII responsiveness using a control FISH probe (e.g., cenFISH), there may be several technical reasons. First, the user should try fresh ExoIII or test the quality of their existing ExoIII. For example, users can incubate 10–25 units of ExoIII with linearized or nicked plasmid DNA. Active ExoIII catalyzes unidirectional digestion of DNA at ∼500 bases per minute at 37°C *in vitro*. Corresponding degradation of the plasmid DNA should be detected when visualized by ethidium bromide-stained agarose gel electrophoresis. Second, we have found that different cell lines respond differently to ExoIII. The ExoIII concentration should therefore be optimized for each cell line. As such, we recommend trying 0, 50, 100, 200, and 500 mU/μL ExoIII in preliminary experiments.

### Problem 4

Positive controls (e.g., Nt.BsmAI / BsmAI) not working (related to STEP 2).

### Potential solution

The user should try fresh Nt.BsmAI / BsmAI when using these restriction enzymes as positive controls. If Nt.BsmAI / BsmAI pre-treatments are still not increasing the FISH signal intensities as expected, this could be due to (a) cell line choice and (b) restriction enzyme amounts. First, the user should ensure that the BsmAI recognition sites are present in the cell line that they are using. For hTERT-RPE1 cells in which this protocol was optimized, BsmAI / Nt.BsmAI recognition sites are every ∼900 bp in human centromeres, on average. To determine the density and distribution of recognition sites in a given region of interest, users can extract the sequence of their region of interest from the appropriate reference genome and identify recognition sites using any standard cloning software (e.g., SnapGene was used in our case). Second, the user can try titrating BsmAI / Nt.BsmAI amounts (e.g., 0 U, 1 U, 2.5 U, 5 U, 10 U, etc.).

## Resource availability

### Lead contact

Further information and requests for resources and reagents should be directed to and will be fulfilled by the lead contact, Fumiko Esashi (fumiko.esashi@path.ox.ac.uk).

### Materials availability

This study did not generate new and unique reagents.

## Data Availability

Original data for figures in the paper is available at Mendeley Data: https://doi.org/10.17632/65jt7xwr2p.1.
